# Genome-wide survey of the seagrass *Zostera muelleri* suggests modification of the ethylene signalling network

**DOI:** 10.1093/jxb/eru510

**Published:** 2015-01-06

**Authors:** Agnieszka A. Golicz, Martin Schliep, Huey Tyng Lee, Anthony W.D. Larkum, Rudy Dolferus, Jacqueline Batley, Chon-Kit Kenneth Chan, Gaurav Sablok, Peter J. Ralph, David Edwards

**Affiliations:** ^1^School of Agriculture and Food Sciences, University of Queensland, Brisbane, QLD 4072,Australia; ^2^Australian Centre for Plant Functional Genomics, School of Land, Crop and Food Sciences, University of Queensland, Brisbane, QLD 4067, Australia; ^3^Plant Functional Biology and Climate Change Cluster, University of Technology Sydney,Ultimo, NSW 2007, Australia; ^4^CSIRO Agriculture Flagship, GPO Box 1600, Canberra ACT 2601, Australia; ^5^School of Plant Biology, University of Western Australia, WA, 6009, Australia

**Keywords:** Ethylene biosynthesis/signalling, gene loss, genome survey, seagrass, *Zostera muelleri*.

## Abstract

Seagrasses are flowering plants which grow fully submerged in the marine environment. They have evolved a range of adaptations to environmental challenges including light attenuation through water, the physical stress of wave action and tidal currents, high concentrations of salt, oxygen deficiency in marine sediment, and water-borne pollination. Although, seagrasses are a key stone species of the costal ecosystems, many questions regarding seagrass biology and evolution remain unanswered. Genome sequence data for the widespread Australian seagrass species *Zostera muelleri* were generated and the unassembled data were compared with the annotated genes of five sequenced plant species (*Arabidopsis thaliana*, *Oryza sativa*, *Phoenix dactylifera*, *Musa acuminata*, and *Spirodela polyrhiza*). Genes which are conserved between *Z. muelleri* and the five plant species were identified, together with genes that have been lost in *Z. muelleri*. The effect of gene loss on biological processes was assessed on the gene ontology classification level. Gene loss in *Z. muelleri* appears to influence some core biological processes such as ethylene biosynthesis. This study provides a foundation for further studies of seagrass evolution as well as the hormonal regulation of plant growth and development.

## Highlight

An aquatic life genome sequencing suggests a complete loss of genes for ethylene biosynthesis and signalling pathways in the seagrasses, *Zostera muelleri* and *Zostera marina*, a new model for hormone studies.

## Introduction

Seagrasses are a polyphyletic group of monocotyledonous plants which are capable of living underwater in marine environments (Larkum *et al.*, 2006; Waycott *et al.*, 2006). They are descendants of terrestrial plants which returned to life in the aquatic environment and consist of ~60 species, most of which have long narrow leaves and grow in large submerged meadows (Wissler *et al.*, 2011). All seagrasses belong to the order Alismatales, which also includes several terrestrial plant species, 11 families of aquatic freshwater species, and four families that are fully marine. The marine families include Posidoniaceae, Zosteraceae, Hydrocharitaceae, and Cymodoceaceae (Larkum *et al.*, 2006; Waycott *et al.*, 2006).

Living fully submerged in a marine environment poses many challenges, including light attenuation through the water column (Dalla Via *et al.*, 1998), resisting the forces of wave action and tidal currents (Waycott *et al.*, 2006), high concentrations of salt in the surrounding seawater, and growing in anoxic marine sediment rich in sulphide (Terrados *et al.*, 1999). Seagrasses have evolved to grow and reproduce under these difficult environmental conditions, emphasizing a unique/novel morphology, physiology, and biochemistry compared with terrestrial plants. They have developed aerenchyma in their leaves, roots, and rhizomes to tolerate anoxia; stomata are absent; and they have reduced stamen and corolla and elongated pollen without an exine layer to facilitate hydrophilous pollination. Living in a saline environment, they have adapted to elevated ionic pressure, as well as high sodium and chloride concentrations. They can take up nitrate from the surrounding water and ammonia from the sediment, and survive in a reducing/anoxic rhizosphere with high levels of sulphides. Many of the fundamental evolutionary questions involving the unique biology of seagrasses remain open, including adaptation to living in a saline environment and the evolution of stress response strategies (Waycott *et al.*, 2006). The identification of seagrass genes and their related cellular processes may provide insights into seagrass evolution and adaptation to life in the marine realm. The characterization of genes that are lost in seagrass compared with other plant species suggests which molecular processes are no longer active or have significantly diverged in seagrass, while the identification of conserved genes between seagrasses and other plants can help resolve processes that are indispensable to plant life both on land and in the marine realm.

Previous genomic analysis of seagrasses has been performed using expressed sequence tags (ESTs) (Reusch *et al.*, 2008; Franssen *et al.*, 2011, 2014; Wissler *et al.*, 2011; Gu *et al.*, 2012; Kong *et al.*, 2013). Recent years have seen major advances in DNA sequence technologies. Many major species now have draft genome assemblies, and DNA sequencing data are providing valuable insights into plant physiology and evolution. Draft genome assembly projects remain expensive and time consuming, and are unlikely to be undertaken for many of the non-model species for some time. However, methods employing the analysis of unassembled sequence data still permit valuable comparative genomic analysis without the expense or time required for whole-genome assembly. In this study, a comparative genomics approach using unassembled whole-genome shotgun sequence data has been applied to characterize gene conservation and loss in the marine seagrass *Zostera muelleri* in comparison with the genomes of five sequenced plants: *Arabidopsis thaliana*, *Oryza sativa* (rice), *Musa acuminata* (banana), *Phoenix dactylifera* (date palm), and *Spirodela polyrhiza* (duckweed). Through the identification of genes that are either lost in *Z. muelleri* or remain conserved, insights can be gained into *Z. muelleri* evolution and adaptation to the marine environment.

The results obtained suggest loss of ethylene biosynthesis and signalling pathways in *Z. muelleri*, which is supported by the analysis of data for *Zostera marina* and *Zostera noltii*, together suggesting loss of ethylene production across the Zosteraceae. Ethylene is a gaseous hormone, a core regulator of many developmental processes, and the ethylene signalling network was thought to be ubiquitously present throughout higher plants (Lin *et al.*, 2009). Seagrasses belonging to the genus *Zostera* present a new model for the study of ethylene signalling and hormone network signalling plasticity, and further studies may provide insights into the physiological and biochemical impacts of life without ethylene.

## Materials and methods

### Genome sequence data

*Zostera muelleri* ssp. *capricorni* (Jacobs *et al.*, 2006) plants were collected from Pelican Banks in Gladstone Harbour (Queensland, Australia) in November 2011, transferred with rhizomes attached in a 5–10cm deep sediment layer into 1 litre rectangular, clear plastic food storage containers, and delivered on the same day to the University of Technology Sydney. The plants were acclimatized for 2 months in an aerated and temperature-controlled mesocosm under Sydney summer conditions with weekly partial seawater changes. Leaf blades of a single plant were snap-frozen and stored in liquid nitrogen for later stage DNA extraction with a soil sample DNA extraction kit (http://www.mobio.com/soil-dna-isolation/powersoil-dna-isolation-kit.html) according to the manufacturer’s instructions. Prior to DNA extraction, the plant material was ground to a fine powder in liquid nitrogen with a pre-chilled mortar and pestle. Genomic DNA was sequenced using an Illumina HiSeq 2000-SBS v3.0 sequencer with 100bp paired-end (PE) technology and an insert size of 304bp at the Ramaciotti Centre at the University of New South Wales (UNSW; NCBI:PRJNA253152). The libraries for genome sequencing were prepared with the Illumina Tru-seq DNA-seq kit.

*Zostera marina* sequencing reads were downloaded from the NCBI Short Read Archive (SRA: SRR494397). A subset of the reads (PE, 140bp in length) totalling ~30× coverage was used for analysis. Furthermore, the coding sequences (CDSs, primary transcript) for *A. thaliana* (TAIR 10; Arabidopsis Genome Initiative, 2000), *O. sativa* (MSU v7; International Rice Genome Sequencing, 2005), *S. polyrhiza* (v2; Wang *et al.*, 2014), *Solanum lycopersicum* (tomato; ITAG2.3; (Tomato Genome Consortium, 2012), *P. dactylifera* (v3), *M. acuminata* (v1; D’Hont *et al.*, 2012), and *Nelumbo nucifera* (lotus; v2; Ming *et al.*, 2013) were downloaded (the full list of ftp addresses are available in the Supplementary data at *JXB* online).

### Genome size and coverage estimation

Genome sizes of *Z. muelleri* and *Z. marina* were estimated using k-mer analysis (Zhang *et al.*, 2013). K-mer distribution was obtained using Jellyfish v2.0 (Marçais and Kingsford, 2011). The k-mer size used was 21 (Supplementary Figs S1, S2 at *JXB* online). The percentage of bases not sequenced at a given coverage was calculated according to the following probability function: P(Y=y)=C^y^∙e^–C^/y!, y=0 (y is the number of times a base is sequenced, C is coverage) (Lander and Waterman, 1988)

### Short read discontiguous (dc-) megaBLAST mapping evaluation

Two different types of BLAST (BLAST+ v2.28) (Camacho *et al.*, 2009) comparison searches were performed. In the first, simulated tomato whole-genome shotgun sequencing reads were mapped to the *A. thaliana* coding sequences (RvsCDS comparison). In the second, full-length tomato CDSs were compared with *A. thaliana* CDSs. Short read sequence data were simulated using the tomato genome as the reference using wgsim (Li *et al.*, 2009), resulting in read-lengths of 100bp and total coverage of ~15×. Reads were mapped to the *A. thaliana* CDSs using dc-megaBLAST (Ma *et al.*, 2002; Camacho *et al.*, 2009) (e-value 1e-5). Only best hits were retained, and horizontal coverage of CDSs (the fraction of the CDSs which had reads mapped to them) was calculated using a custom python script calculate_blast_coverage.py. CDSs with only one read mapping, or multiple reads mapping, all of them having the same start and end co-ordinates were considered to have horizontal coverage 0.

*Solanum lycopersicum* (tomato) CDSs were compared with *A. thaliana* CDSs using dc-megaBLAST (e-value 1e-5) and only best hits were retained.

The two BLAST results were used to evaluate the feasibility of using short read mapping as replacement for the whole-length CDS comparison. To evaluate the robustness of the proposed approach, discrepancy rates (DRs) between RvsCDS and CDSvsCDS results were calculated according to the following formulae:

DR genes lost [DRL]=(number of genes defined as lost based on horizontal coverage in RvsCDS comparison that had a significant hit in the CDSvsCDS comparison)/(total number of genes defined as lost in RvsCDS comparison)

DR genes conserved [DRC]=(number of genes defined as conserved based on horizontal coverage in RvsCDS comparison that had no significant hit in the CDSvsCDS comparison)/(total number of genes defined as conserved in RvsCDS comparison)

The DR values were plotted against different horizontal coverage cut-off values and used to define optimal horizontal coverage values for further analysis.

### Calculation of CDS coverage

Sequencing reads from *Z. muelleri* and *Z. marina* were compared with the CDSs from five plant species: *A. thaliana*, *O. sativa*, *P. dactylifera*, *M. acuminata*, and *S. polyrhiza* using dc-megaBLAST (e-value 1e-5). Only best hits were retained, and CDS coverage was determined as previously described in the section on short read discontiguous (dc-) megaBLAST mapping evaluation.

### Orthologous gene clusters (OGCs) construction

A set of genes conserved between the seven species (monocot: *O. sativa*, *P. dactylifera*, *M. acuminata*, and *S. polyrhiza*; and dicot: *A. thaliana*, *S. lycopersicum*, and *N. nucifera*) was identified using reciprocal best BLAST (RBB; BLASTP) with an e-value 1e-5 threshold between *A. thaliana* genes and genes from the remaining six species. If an *A. thaliana* gene had more than one RBB hit within the same species (more than one top score in reciprocal searches based on the e-value), bit scores were considered; the gene with the highest bit score and all the genes within 10 points of the highest bit score were included as RBB hits. Genes were assigned into clusters, each containing one *A. thaliana* gene and its putative orthologues in the six remaining species. A subset of OGCs including only clusters which contained at least one gene originating from a monocot species was extracted and termed OGCsM.

### Identification of genes lost and conserved

For each gene in the OGCsM, previously calculated CDS coverages were inspected across *A. thaliana* and all the monocot orthologous genes present in the cluster. If the average coverage across all CDSs was <2%, the cluster was considered to represent a gene lost in seagrass. If the average coverage across all CDSs was >50%, the cluster was considered to represent a gene conserved in seagrass. OGCsM rather the full set of OGCs was considered since *Z. marina* and *Z. muelleri* are monocots and dc-megaBLAST comparison against other monocot species is more likely to be successful. The cut-off values were chosen based on calculated DRs. Each OGCM consisted of at least two genes (the *A. thaliana* gene and at least one orthologue in the monocot species). Therefore, to be considered as a lost or conserved gene it had to be present in *A. thaliana* and at least one of the four remaining species (*O. sativa*, *P. dactylifera*, *M. acuminata*, and *S. polyrhiza*). The approach used allows identification of genes lost and conserved using unassembled genomic data. However, it is important to note that while this approach will identify genes which are lost or conserved relative to the existing reference gene set, it will not discover newly evolved genes.

### Gene ontology annotation

The *A. thaliana* orthologue from each identified cluster was used to derive gene ontology (GO) annotation (Ashburner *et al.*, 2000) available via TAIR (Lamesch *et al.*, 2012) (version available on 7 March 2014, GO terms with NR and ND evidence codes were filtered out). The topGO R package (Alexa *et al.*, 2006) available via Bioconductor (Gentleman *et al.*, 2004) v2.14 was used to test for enrichment of GO annotation terms using all identified OGCsM clusters as background. Enrichment was assessed using the Fisher exact test (using weight count).

### Analysis of ESTs from the Dr Zompo database

ESTs representing the A, B, and C labelled data sets for *Z. marina* in the Dr Zompo database and the A data set for *Z. noltii* were downloaded from the Dr Zompo EST database (Wissler *et al.*, 2009). UCLUST (Edgar, 2010) (identity threshold 0.99) was used to remove redundant ESTs. RBB hits, as described above, were identified between *A. thaliana* CDSs and *Z. marina* and *Z. noltii* ESTs.

### Analysis of ESTs from three terrestrial Alismatales species

Assembled transcriptomes of *Anthurium andraeanum* (Tian *et al.*, 2013) (PRJNA24104) and *Zantedeschia aethiopica* (PRJNA205467) were downloaded from NCBI (http://www.ncbi.nlm.nih.gov/). UCLUST (v7.0; Edgar, 2010; identity threshold 0.99) was used to remove redundant ESTs. RBB hits, as described above, were identified between *A. thaliana* CDSs and ESTs. Unassembled sequencing reads from *Amorphophallus bulbifer* were downloaded from SRA (SRR553186). Genes present were identified using the same strategy as described above for *Z. muelleri* and *Z. marina* genomes. Mirroring the threshold of <2% to consider a gene as lost, a threshold of ≥2% was used to consider a gene as present.

### Multiple sequence alignments of EIN3-like 1 (EIL1) orthologues

Multiple sequence alignment between *Z. marina* and *Z. noltii* EIL1 orthologues, identified from the Dr Zompo EST database, EIN3/EIL1 orthologues in the seven monocot and dicot species identified in OGCs, and EIN3 orthologues identified in *A. andraeanum* and *Z. aethiopica* transriptomes was performed using T-coffee (Notredame *et al.*, 2000) in Jalview (v2.8.1) (Waterhouse *et al.*, 2009). EIL1 and EIN3 were identified based on RBB hits with *A. thaliana* genes. The transcripts corresponding to EIL1 and EIN3 were translated in all six reading frames using EXPASY (http://www.expasy.org/). The longest open reading frame (ORF) was chosen for further analysis.

## Results

### Estimated genome coverage

In this study, whole-genome sequencing of *Z. muelleri* was conducted, yielding a total of 39 Gbp of sequence data (191 141 643 read pairs), with an average coverage of ~43×. Additionally, ~30× genome coverage *Z. marina* sequence data were used for comparative analysis and genome size estimation. The estimated genome size of *Z. muelleri* based on the K-mer distribution (Huang *et al.*, 2009; Potato Genome Sequencing Consortium, 2011; Parkin *et al.*, 2014) is ~900 Mbp, which is around twice the size of the *Z. marina* genome, which is estimated to be 430 Mbp based on the K-mer distribution. With the estimated coverages being 30× and above, all of the two genomes should be sampled according to Lander–Waterman statistics (Lander and Waterman, 1988) (at 30× coverage the faction of the genome that will not be sequenced is 9.4e–12%), so the gene loss presented here is unlikely to be due to uneven sampling of the genome.

### Evaluation of short read mapping as a gene conservation/loss detection tool

**Fig. 1.**
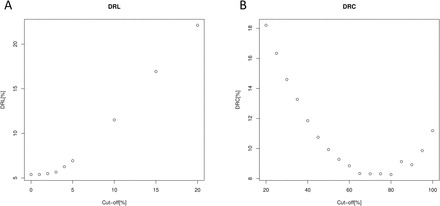
Discrepancy rates (DRs) calculated based on tomato data for genes lost (DRL) (A) and conserved (DRC) (B) using different horizontal coverage cut-offs.

In order to evaluate the feasibility and robustness of using short read mapping as a replacement for full-length CDS comparison as a gene loss/conservation detection tool, two BLAST comparison searches were performed. Tomato was chosen for simulations because of a similar estimated divergence time (~120–130 million years) between tomato and *A. thaliana*, and between Alismatales (which include *Z. muelleri* and *Z. marina*) and core monocots (Janssen and Bremer, 2004; Wang *et al.*, 2014). In the first comparison, simulated tomato whole-genome sequencing reads were mapped against *A. thaliana* CDSs (RvsCDS comparison). In the second comparison, full-length tomato CDSs were compared with *A. thaliana* CDSs (CDSvsCDS comparison). The DRs between RvsCDS and CDSvsCDS search results were calculated using different horizontal coverage cut-off values and are presented in Fig. 1. Based on the DR values, coverage cut-offs were defined as 2% for genes to be considered lost.

### OGC construction and identification of genes lost and conserved

A total of 17 522 OGCs were identified. A total of 16 007 clusters which contained at least one orthologue representing a monocot species (OGCsM) were found. A list of OGCs genes can be found in Supplementary Table S1 at *JXB* online. A total of 4091 genes conserved and 2147 genes lost between *Z. muelleri* and the five other plant species (*A. thaliana*, *O. sativa*, *P. dactylifera*, *M. acuminata*, and *S. polyrhiza*) were identified, respectively. Lists of genes conserved and lost can be found in Supplementary Tables S2 and S3 at *JXB* online, respectively.

### GO analysis of genes conserved suggests conservation of fundamental biological processes

GO analysis identified 175 significantly enriched (*P*<0.05) biological process terms within the conserved gene data set. The 39 GO terms with the lowest *P*-values are presented in Table 1, and a full list can be found in Supplementay Table S4 at *JXB* online. Biological processes significantly enriched in genes conserved include: glucose metabolic processes, ribosome biogenesis, translation, photosynthesis, and response to salt stress.

**Table 1.** Top 39 significantly enriched GO terms corresponding to the genes predicted to be conserved in Z. muelleri compared with five other plant species and the corresponding P-values

GO ID	Term	*P*-value
GO:0006091	Generation of precursor metabolites and energy	<1e-30
GO:0006094	Gluconeogenesis	<1e-30
GO:0046686	Response to cadmium ion	<1e-30
GO:0006412	Translation	<1e-30
GO:0019320	Hexose catabolic process	<1e-30
GO:0010498	Proteasomal protein catabolic process	<1e-30
GO:0070271	Protein complex biogenesis	<1e-30
GO:0051788	Response to misfolded protein	3.80E-22
GO:0016192	Vesicle-mediated transport	4.30E-21
GO:0015979	Photosynthesis	1.20E-20
GO:1901605	α-Amino acid metabolic process	2.20E-19
GO:0043094	Cellular metabolic compound salvage	9.90E-19
GO:0007030	Golgi organization	6.40E-17
GO:0009651	Response to salt stress	7.30E-17
GO:0019941	Modification-dependent protein catabolic process	1.20E-16
GO:0046034	ATP metabolic process	3.30E-16
GO:0006195	Purine nucleotide catabolic process	3.80E-16
GO:0009207	Purine ribonucleoside triphosphate catabolic process	8.80E-16
GO:0015991	ATP hydrolysis-coupled proton transport	2.10E-15
GO:0001510	RNA methylation	5.30E-15
GO:0009225	Nucleotide-sugar metabolic process	1.10E-14
GO:0008104	Protein localization	1.20E-14
GO:0042254	Ribosome biogenesis	1.30E-14
GO:0007264	Small GTPase-mediated signal transduction	1.60E-14
GO:0015748	Organophosphate ester transport	3.30E-14
GO:0043648	Dicarboxylic acid metabolic process	6.20E-13
GO:0046129	Purine ribonucleoside biosynthetic process	6.90E-13
GO:0006354	DNA-templated transcription, elongation	1.20E-12
GO:0007010	Cytoskeleton organization	2.20E-12
GO:0080147	Root hair cell development	3.00E-12
GO:0051645	Golgi localization	1.40E-11
GO:0051646	Mitochondrion localization	1.40E-11
GO:0060151	Peroxisome localization	1.40E-11
GO:1901293	Nucleoside phosphate biosynthetic process	2.70E-11
GO:0006833	Water transport	2.20E-10
GO:0051273	β-Glucan metabolic process	6.50E-10
GO:0009240	Isopentenyl diphosphate biosynthetic process	1.50E-08
GO:0006779	Porphyrin-containing compound biosynthetic process	1.80E-08
GO:0006740	NADPH regeneration	2.30E-08

### GO analysis of genes lost in *Z. muelleri* points to modification of ethylene biosynthesis and signalling

The list of *A. thaliana* representatives of genes lost in *Z. muelleri* was tested for over-representation of GO terms. In total, 28 GO terms in the biological process category were significantly over-represented (*P*<0.05). The GO terms and associated confidence values are presented in Table 2. The biological processes most influenced by gene loss include lipid transport, ethylene biosynthesis and signalling, response to iron ion, and defence responses. Out of 78 genes in the OGCsM which are associated with ethylene biosynthesis, 23 are lost in *Z. muelleri*. Additionally out of 10 genes involved in the regulation of ethylene-mediated signalling, six are lost in *Z. muelleri*.

**Table 2.** Significantly enriched GO terms corresponding to the genes predicted to be lost in *Z. muelleri*

GO ID	Term	*P*-value
GO:0006869	Lipid transport	2.00E-09
GO:0009693	Ethylene biosynthetic process	7.20E-07
GO:0042218	1-Aminocyclopropane-1-carboxylate biosynthetic process	8.60E-06
GO:0010105	Negative regulation of ethylene-activated signalling pathway	0.00012
GO:0009807	Lignin biosynthetic process	0.00015
GO:0010044	Response to aluminium ion	0.00041
GO:0071281	Cellular response to iron ion	0.0007
GO:0048451	Petal formation	0.00085
GO:0048453	Sepal formation	0.00085
GO:0002237	Response to molecule of bacterial origin	0.00102
GO:0080027	Response to herbivore	0.00112
GO:0010227	Floral organ abscission	0.00135
GO:0009813	Flavonoid biosynthetic process	0.00161
GO:0009626	Plant-type hypersensitive response	0.0029
GO:0009408	Response to heat	0.00345
GO:0052544	Defence response by callose deposition in cell wall	0.00374
GO:0006952	Defence response	0.00504
GO:0042542	Response to hydrogen peroxide	0.00514
GO:0015824	Proline transport	0.00848
GO:0010941	Regulation of cell death	0.01416
GO:0009625	Response to insect	0.01477
GO:0005983	Starch catabolic process	0.01486
GO:0001101	Response to acid	0.01569
GO:0009611	Response to wounding	0.01739
GO:0009736	Cytokinin-activated signalling pathway	0.0264
GO:2000038	Regulation of stomatal complex development	0.03496
GO:0036294	Cellular response to decreased oxygen levels	0.04875
GO:0010375	Stomatal complex patterning	0.04875

This enrichment analysis suggests that ethylene biosynthesis and signalling is highly impacted by gene loss in *Z. muelleri*. Therefore, a detailed analysis was performed on 27 core genes associated with ethylene biosynthesis and signalling (Table 3).

**Table 3.** Summary of 27 genes involved in ethylene synthesis and signalling which were used in the analysisThree categories are possible: gene present (+), gene absent (–), and information not available (N/A).

Gene ID	Protein name	Function	OGCsM	*Z. muelleri* genome	*Z. marina* genome	*Z. marina* ESTs	*Z. noltii* ESTs
*AT1G12010*	ACO	ACC oxidase	–	N/A	N/A	–	–
*AT2G19590*	ACO1	ACC oxidase	+	–	–	–	–
*AT1G62380*	ACO2	ACC oxidase	+	–	–	–	–
*AT1G05010*	ACO4	ACC oxidase	+	–	–	–	–
*AT1G77330*	ACO5	ACC oxidase	+	–	–	–	–
*AT3G61510*	ACS1	ACC synthase	+	–	–	–	–
*AT1G01480*	ACS2	ACC synthase	+	–	–	–	–
*AT2G22810*	ACS4	ACC synthase	+	–	–	–	–
*AT5G65800*	ACS5	ACC synthase	+	–	–	–	–
*AT4G11280*	ACS6	ACC synthase	+	–	–	–	–
*AT4G26200*	ACS7	ACC synthase	+	–	–	–	–
*AT4G37770*	ACS8	ACC synthase	+	–	–	–	–
*AT3G49700*	ACS9	ACC synthase	+	–	–	–	–
*AT1G62960*	ACS10	No ACC synthase activity	+	+	+	+	+
*AT4G08040*	ACS11	ACC synthase	+	–	–	–	–
*AT5G51690*	ACS12	No ACC synthase activity	+	+	+	–	–
*AT2G40940*	ERS1	Ethylene receptor	+	–	–	–	–
*AT1G04310*	ERS2	Ethylene receptor	–	N/A	N/A	–	–
*AT1G66340*	ETR1	Ethylene receptor	+	–	–	–	–
*AT3G23150*	ETR2	Ethylene receptor	+	–	–	–	–
*AT3G04580*	EIN4	Ethylene receptor	+	–	–	–	–
*AT5G03730*	CTR1	Raf-like kinase	+	+	–	–	–
*AT5G03280*	EIN2	Signal transducer	+	–	–	–	–
*AT3G20770*	EIN3	Transcription factor	+	+	+	–	–
*AT2G27050*	EIL1	Transcription factor	+	+	+	+	+
*AT5G21120*	EIL2	Transcription factor	–	N/A	N/A	–	–
*AT1G73730*	EIL3	Transcription factor	+	+	+	+	–

### The *S. polyrhiza* genome and trancriptomes of three terrestrial species belonging to the order Alismatales contain genes associated with ethylene biosynthesis

*Spirodela polyrhiza* is a close relative of seagrasses (both belong to the order Alismatales) and its genome was recently sequenced (Wang *et al.*, 2014). It was postulated that another member of the genus *Spirodela*, *S. oligorrhiza*, produces ethylene under stress conditions via an alternative pathway without the involvement of 1-aminocyclopropane-1-carboxylic acid (ACC) synthase (ACS) and ACC oxidase (ACO) (Mattoo *et al.*, 1992). However, an *S. polyrhiza* orthologue of both ACS (*Spipo24G0002100*) and ACO (*Spipo23G0011700*) was identified in the OGCsM. Additionally, orthologues of ethylene receptors (*Spipo6G0049300* and *Spipo1G0021500*), CTR1 (*Spipo0G0009700*), and EIN2 (*Spipo8G0029200*) were also identified in *S. polyrhiza*.

Genes associated with ethylene synthesis and signalling are present in the transcriptomes of three terrestrial Alismatales species: *A. andraeanum*, *Z. aethiopica*, and *A. bulbifer*. In *A. andraeanum*, transcripts corresponding to orthologues of ACO, ethylene receptors, EIN2, and EIN3 were detected. In *Z. aethiopica*, transcripts corresponding to orthologues of ACO, ethylene receptors, CTR1, EIN2, EIN3, and EIL3 were found, while in *A. bulbifer* transcripts corresponding to orthologues of ACO, ACS, ethylene receptors, CTR1, EIN2, EIN3, EIL1, and EIL3 could be detected.

### Multiple genes involved in ethylene biosynthesis and signalling are lost in *Z. muelleri* and *Z. marina*

Genes lost in *Z. muelleri* include all orthologues of the genes found in OGCsM encoding ACS (encoding orthologues of: ACS1, ACS2, ACS4, ACS5, ACS6, ACS7, ACS8, ACS9, and ACS11) and all orthologues of the genes found in OGCsM encoding ACO (encoding orthologues of: ACO1, ACO2, ACO4, and ACO5). Additionally, orthologues of OGCsM genes associated with the regulation of ethylene signalling are lost in *Z. muelleri*. The list includes orthologues of four well characterized ethylene signalling molecules (ETR1, ETR2, ERS1, and EIN4). The orthologue of an ethylene signal transducer protein EIN2 is also missing in *Z. muelleri*. Orthologues of transcription factors EIN3, EIL1, and EIL3 appear to be present in *Z. muelleri* (Table 3).

Using the same read mapping method, the presence of orthologues of the genes listed in Table 3 was tested for in the genome of another seagrass species, *Z. marina*. The genes encoding orthologues of ACS1, ACS2, ACS4, ACS5, ACS6, ACS7, ACS8, ACS9, ACS11, ACO1, ACO2, ACO4, ACO5, ETR1, ETR2, ERS1, EIN4, CTR1, and EIN2 also appear to be absent in the *Z. marina* genome.

### Analysis of ESTs supports loss of ethylene biosynthesis and signalling in *Z. marina* and *Z. noltii*

RBB searches between *A. thaliana* CDSs and *Z. marina* ESTs revealed 10 074 RBB relationships in total. The only transcripts corresponding to proteins involved in ethylene biosynthesis and signalling that were identified in the *Z. marina* EST database correspond to the orthologues of EIL1 and EIL3, similar to the observations in *Z. muelleri* (Table 3). Similarly, RBB searches between *A. thaliana* CDSs and *Z. noltii* ESTs resulted in 8793 RBB relationships, and the only transcript corresponding to proteins involved in ethylene biosynthesis and signalling encodes a putative orthologue of EIL1.

### Multiple sequence alignment between *Z. marina* EIL1 and orthologues of EIL1 and EIN3 found in OGCs suggests at least partial conservation of function

Multiple sequence alignment between *Z. marina* EIL1, orthologues of EIL1 and EIN3 found in OGCs, and EIN3 orthologues found in *A. andraeanum* and *Z. aethiopica* transriptomes was performed. Both EIL1 and EIN3 are homologous proteins ~600 amino acids in length, containing three domains: a DNA-binding domain (DBD) at the N-terminus, a proline-rich domain (PRD) in the central region, and a protein degradation domain (PDD) at the C-terminus (Cho *et al.*, 2012). The DBD, which spans five α-helices (Yamasaki *et al.*, 2005), appears to be well conserved in *Z. marina* (Supplementary Fig. S3 at *JXB* online). The two key DNA binding residues Pro216 and Lys245 (Cho *et al.*, 2012) are also conserved. However, both *Z. marina* and *Z. noltii* EIL1 orthologues share an N-terminal deletion of ~130 residues.

## Discussion

Whole-genome shotgun sequence data for the seagrass species *Z. muelleri* was compared with gene sets of four sequenced terrestrial species, *A. thaliana*, *O. sativa*, *P. dactylifera*, and *M. acuminata*, and one aquatic species *S. polyrhiza*. The species list contains a model dicot and a collection of four monocots. The genome-wide analysis of *Z. muelleri* gene conservation and loss aimed to identify genes which are common between these species as well as genes which are lost in *Z. muelleri*. The loss of ethylene biosynthesis and signalling genes in *Z. muelleri* was supported by the analysis of the *Z. marina* genome and transcriptome.

The genes which were identified as conserved between the five species and *Z. muelleri* are involved in a range of highly conserved cellular processes. The processes with the most confident GO term enrichment values include glucose metabolism, protein catabolism, and translation, and many of the genes that are involved in carbohydrate and protein metabolism. It is clear that many of the basic cellular processes are conserved between *Z. muelleri* and other plant species. The conservation of genes involved in response to salt stress also suggests that *Z. muelleri* may use some of the same pathways to combat salinity in the marine environment as other plant species.

The analysis of genes lost suggests major disruption of ethylene synthesis and signalling in *Z. muelleri* and *Z. marina*. Ethylene is a simple two-carbon gaseous compound which is a potent modulator of plant growth and development (Ecker, 1995). This plant hormone is involved in many aspects of the plant life cycle, including seed germination, root hair development, root nodulation in legumes, flower senescence, abscission, and fruit ripening (Johnson and Ecker, 1998; Wang *et al.*, 2002). The production of ethylene is known to be controlled by internal signals during development and in response to external abiotic/biotic stimuli (Wang *et al.*, 2002) including: wounding, drought, ozone, flooding, pathogen and insect attack, and salt stress (Cao *et al.*, 2007, 2008; Wang *et al.*, 2007; Yoo *et al.*, 2009).

**Fig. 2.**
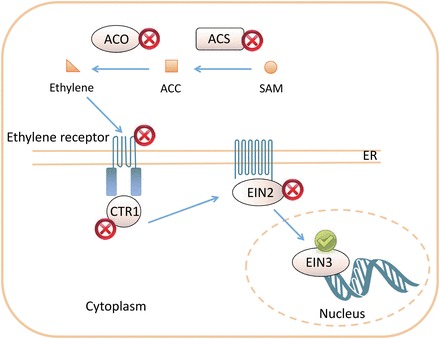
Ethylene biosynthesis and signalling. Ethylene is synthesized from *S*-adenosylmethionine (SAM) by ACC (1-aminocyclopropane-1-carboxylic acid) synthase (ACS) and ACC oxidase (ACO) to form ethylene. Ethylene is perceived by a range of receptors and the signal is further transduced by kinase CTR1 and EIN2. A family of EIN3 transcription factors are activated by ethylene. Proteins marked with a cross are most probably lost in *Z. muelleri*. Proteins marked with a tick are most probably present.

Ethylene is synthesized from *S*-adenosylmethionine (SAM). SAM is converted to ACC by ACS, and ACC is then oxidized by ACO to form ethylene (Wang *et al.*, 2002). Ethylene is perceived by a range of receptors, including the five receptors found in *A. thaliana*: ETR1, ETR2, EIN4, ERS1, and ERS2. The ethylene signal is further transduced by a Raf-like kinase CTR1 and finally EIN2, which can activate a family of EIN3 transcription factors (Wang *et al.*, 2002). The present analysis suggests that ethylene metabolism and signalling functions are missing in *Z. muelleri*, and that there is a complete loss of ethylene biosynthesis and signalling in this species (Fig. 2).

The loss of ethylene synthesis and signalling in Zosteraceae is further supported by analysis of *Z. marina* and *Z. noltii* EST data available in the Dr Zompo database (Wissler *et al.*, 2009). Genes involved in ethylene synthesis and signalling are ubiquitously expressed in many plant species (Wang *et al.*, 2002). Therefore, if they were present in the *Z. marina* genome, at least some of them may be expected to be expressed and present in the EST database.

In terrestrial plants, ethylene has been shown to be implicated in response to flooding stress (Voesenek and Sasidharan, 2013), such as triggering programmed cell death that leads to the formation of aerenchyma, a tissue which assists in conducting oxygen to the submerged root system of the plant (Voesenek *et al.*, 2006). Aerenchyma in seagrasses are present constitutively in leaves and roots as an adaptation to a submerged life, which in turn might supersede any requirement for ethylene signalling for aerenchyma formation. Life on the sea floor is also associated with qualitative and quantitative changes in available light. In many plants, low-light conditions normally cause etiolation, shade avoidance, and leaf senescence, responses that involve ethylene (Franklin, 2008). Many seagrasses are adapted to low-light conditions (Ralph *et al.*, 2007), again possibly obviating the need for ethylene in inducing shade avoidance responses. Ethylene is also constantly emitted by plants in a range of physiological processes such as during the circadian rhythm of the plant, during flower development, or upon wounding stress or in response to environmental stressors (Harren and Cristescu, 2013). In turn, ectopic ethylene induces ethylene signalling pathways within the plant (Barry *et al.*, 2005; Liu and Wen, 2012). Hence, a plausible explanation as to why ethylene could be lost in plants under submerged marine conditions might be the drastically reduced efficacy of a gaseous hormone such as ethylene in an aquatic environment. Analysis of the genome and transcriptomes of four diverse species belonging to the order Alismatales suggests the presence of ethylene synthesis and signalling in all four species. It was postulated that *S. oligorrhiza*, a close relative of *S. polyrhiza*, does not produce ethylene via the ACC intermediate but via an alternative pathway (Mattoo *et al.*, 1992). However, in this study, genes which appear to be orthologues of ACS and ACO were identified in the *S. polyrhiza* genome. Furthermore, *S. polyrhiza* possesses many components of the ethylene signalling pathway which appear to be lost in *Z. muelleri* and *Z. marina*. The presence of ethylene signalling in *S. polyrhiza*, which grows forming mats on the water surface, and its absence in the two seagrasses, *Z. muelleri* and *Z. marina*, which grow fully submerged, suggests that the loss of ethylene signalling may be associated with a fully submerged environment. However, additional analysis including studies of more aquatic species is necessary to test this hypothesis.

Analysis of both genomic and transcriptomic data points to the conservation of some of the downstream targets of the ethylene signalling cascade in seagrasses. Orthologues of EIN3/EIL transcription factors appear to be present in *Z. muelleri*, *Z. marina*, and *Z. noltii*. Interestingly, the genes encoding EIL1 putative orthologues in *Zostera* (the only detected transcript that could encode a protein involved in ethylene biosynthesis and signalling) have a large deletion in the N-terminal region (~130 amino acids). It could be hypothesized that the loss of the ethylene signalling network has relaxed the selective pressure of all genes involved in this pathway (considering that they are not involved in other functions). This could have offered the opportunity to recruit some of these genes in new functions. Such genes could have been under transient positive selection favouring amino acid changes or deletions (such as the loss of the N-terminal part of EIL1). Further analysis of these transcription factors, their sequences, regulators, and downstream targets may shed more light on the effects of loss of ethylene signalling.

## Supplementary data

Supplementary data are available at *JXB* online.

Figure S1. K-mer distribution for the *Z. muelleri* genome.

Figure S2. K-mer distribution for the *Z. marina* genome.

Figure S3. EIL1 multiple sequence alignment.

Table S1. Complete list of genes found in OGCs.

Table S2. Complete list of genes conserved with corresponding GO annotation.

Table S3. Complete list of genes lost with corresponding GO annotation.

Table S4. Complete list of significantly enriched GO terms associated with genes conserved in *Z. muelleri*.

Supplementary Data
