# Supplementary material for: Genome-wide survey of the seagrass Zostera muelleri suggests modification of the ethylene signalling network
Source: J Exp Bot. 2015 Jan 6;66(5):1489–98. doi: 10.1093/jxb/eru510 (PMC4339605; doi:10.1093/jxb/eru510)
Supplement: Supplementary Data [file supp_66_5_1489__index.html]

Genome-wide survey of the seagrass Zostera muelleri suggests modification of the ethylene signalling network — Supplementary Data 

# Genome-wide survey of the seagrass *Zostera muelleri* suggests modification of the ethylene signalling network

## Supplementary Data

Data files

**Files in this Data Supplement:**

- Supplementary Data - Supplementary Data
- Supplementary Data - Supplementary Data
