# Supplementary material for: Genome-wide survey of the seagrass Zostera muelleri suggests modification of the ethylene signalling network
Source: J Exp Bot. 2015 Jan 6;66(5):1489–98. doi: 10.1093/jxb/eru510 (PMC4339605; doi:10.1093/jxb/eru510)
Supplement: Supplementary Data [file supp_eru510_SupplementaryMaterial1_good2_updated.pdf]

Supplementary Material

Genome-wide survey of the seagrass *Zostera muelleri* suggests modification of the ethylene signalling network

Authors: Agnieszka A Golicz, Martin Schliep, Huey Tyng Lee, Anthony WD Larkum, Rudy Dolferus, Jacqueline Batley, Chon-Kit Kenneth Chan, Gaurav Sablok, Peter J Ralph, David Edwards.

Coding sequence download ftp sites:

A.thaliana, *S. lycopersicum*, *O. sativa*, *S. polyrhiza* – Phytozome v10 (phytozome.jgi.doe.gov)

P.dactylifera - http://qatar-weill.cornell.edu/research/datepalmGenome/download.html

M. acuminata - http://banana-genome.cirad.fr/content/download-dh-pahang

N. nucifera - https://genomevolution.org/CoGe/GenomeInfo.pl?gid=16884

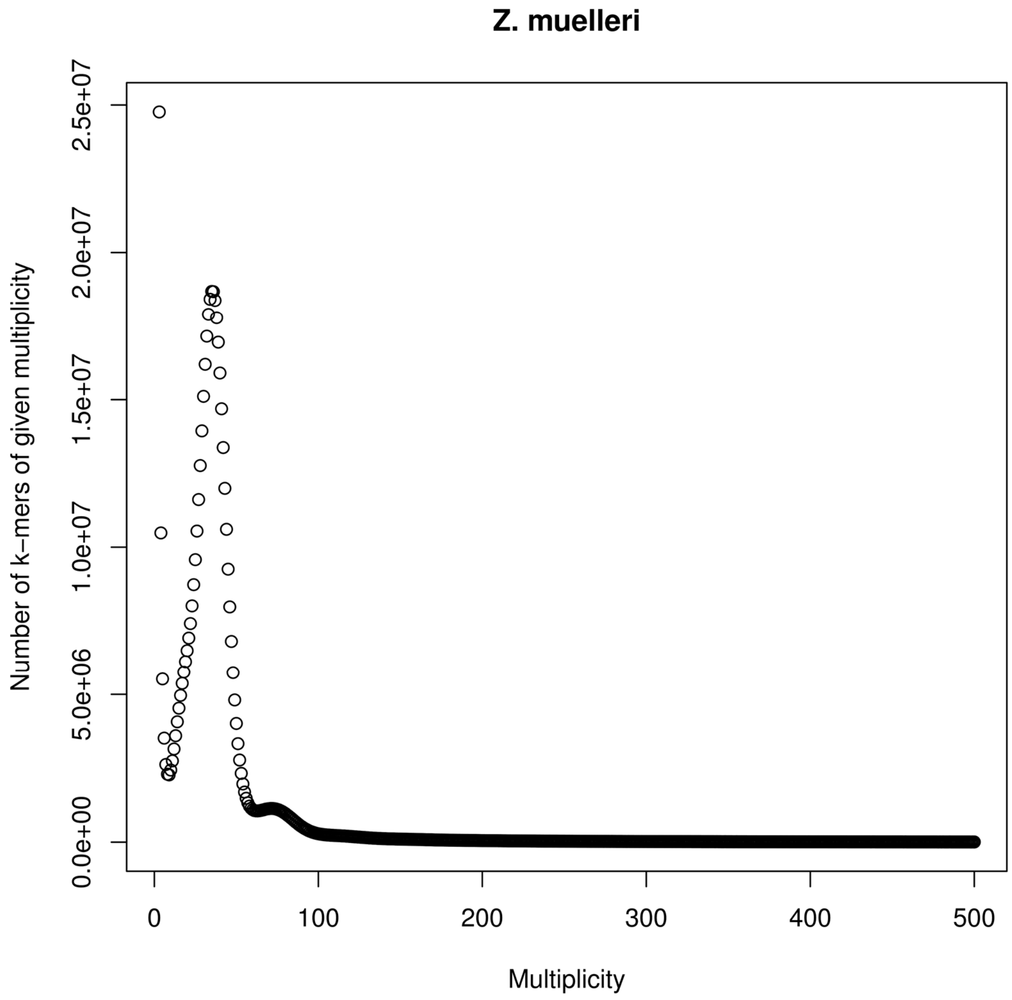

Fig S1. K-mer distribution for *Z. muelleri* genome. K-mers with multiplicity one and two were removed for image clarity.

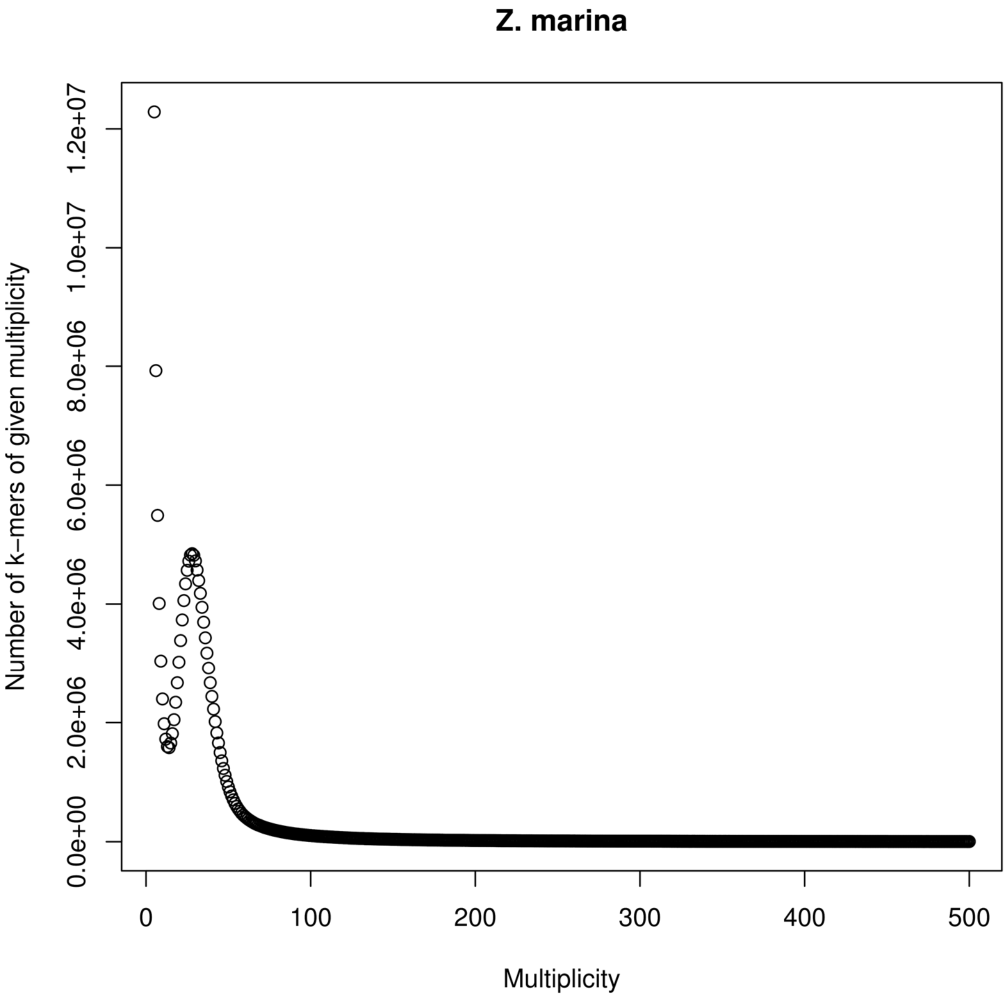

Fig S2. K-mer distribution for *Z. marina* genome. K-mers with multiplicity one to four were removed for image clarity.

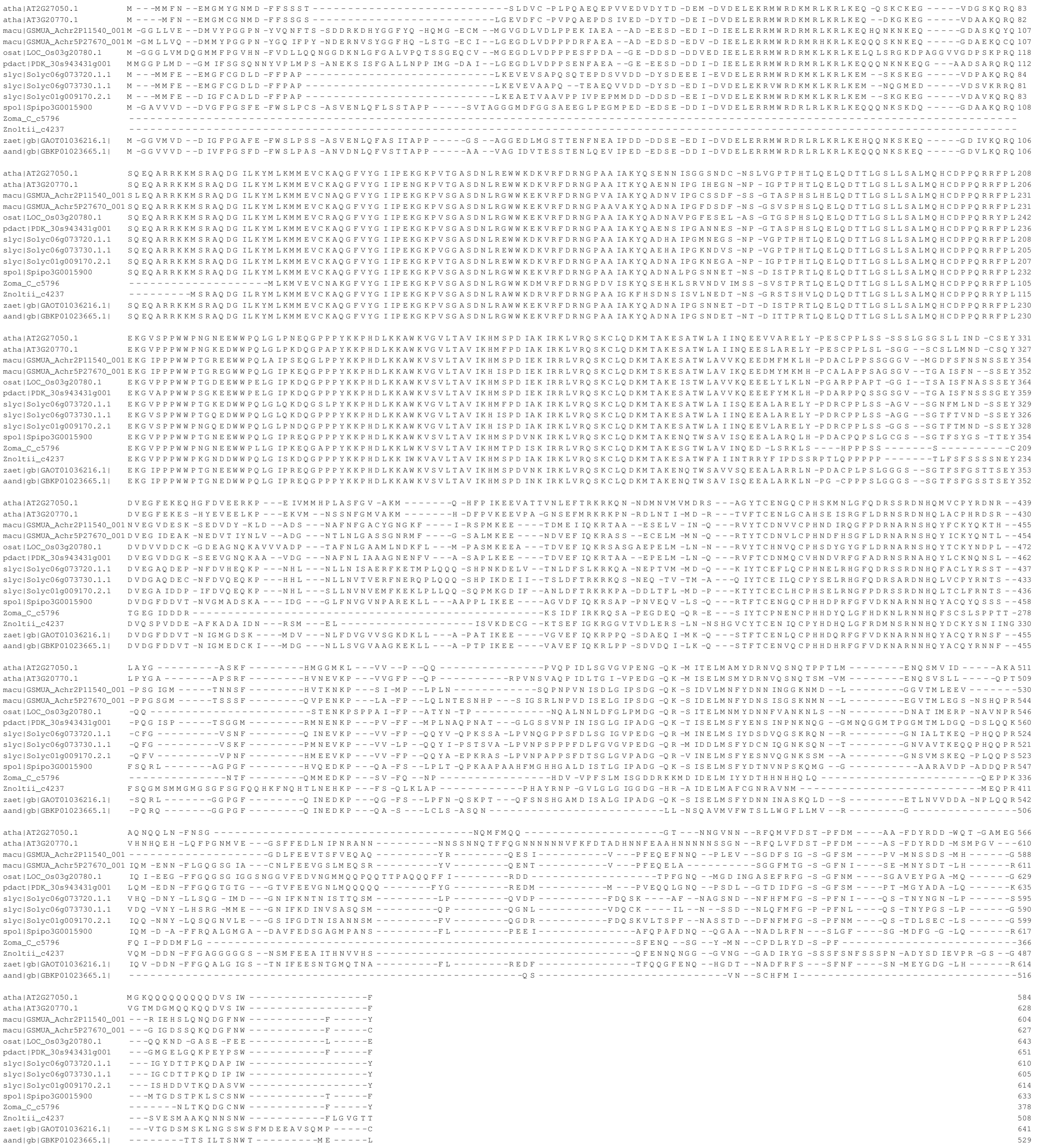

Fig S3. EIL1 multiple sequence alignment. Multiple sequence alignment between *Z. marina* EIL1, *Z. noltii* EIL 1 and EIN3/EIL1 putative orthologs found in OGCs (atha – *A. thaliana*, slyc – S. *lycopersicum*, osat – *O. sativa*, pdact – *P. dactylifera*, macu – *M. acuminata*, spol – *S. polyrhiza*, zaet - *Z. aethiopica*, aand - *A. andraeanum*, Zoma – *Z. marina*, Znoltii – *Z. noltii*).
